# Desmosomes: a role in cancer?

**DOI:** 10.1038/sj.bjc.6603808

**Published:** 2007-05-22

**Authors:** M Chidgey, C Dawson

**Affiliations:** 1 Division of Medical Sciences, University of Birmingham, Clinical Research Block, Queen Elizabeth Hospital, Birmingham B15 2TH, UK; 2 Cancer Research UK Institute for Cancer Studies, University of Birmingham, Vincent Drive, Edgbaston, Birmingham B15 2TT, UK

**Keywords:** desmosome, desmocollin, desmoglein, plakoglobin, EMT

## Abstract

Much evidence now attests to the importance of desmosomes and their constituents in cancer. Alterations in the expression of desmosomal components could contribute to the progression of the disease by modifying intracellular signal transduction pathways and/or by causing reduced cell adhesion. The Wnt/*β*-catenin pathway is a potential target because of the involvement of the cytoplasmic desmosomal protein plakoglobin. Loss of desmosomal adhesion is a prerequisite for the epithelial–mesenchymal transition, implicated in the conversion of early stage tumours to invasive cancers.

Desmosomes are intercellular junctions that confer strong cell–cell adhesion. They are found in epithelia and cardiac muscle and are located at the cell membrane, where they act as anchors for intermediate filaments. Mutations in genes encoding desmosomal constituents can have devastating effects on tissue integrity, but it would be a mistake to assume that desmosomes are simply static adhesive structures; much evidence now indicates that they play an important part in the regulation of cell proliferation and differentiation. Furthermore, there is a strong possibility that desmosomes influence epithelial cell invasion and metastasis. The intention of this review is to concentrate on the emerging evidence that implicates desmosomes in cancer. Other substantive reviews that deal with desmosomes and their role in other human diseases are available (Getsios *et al*, 2004; Kottke *et al*, 2006).

## DESMOSOME COMPOSITION

Desmosomes are made of proteins that belong to one of the three gene families, the desmosomal cadherins, the armadillo family and the plakin family of cytolinkers.

### Desmosomal cadherins

The desmosomal cadherins are the membrane-spanning constituents of desmosomes, and in humans there are seven, three desmocollins (Dsc1–3) and four desmogleins (Dsg1–4). All desmosomes contain at least one desmocollin and one desmoglein and both are required for adhesion. Each of the desmocollins exists as a pair of proteins generated by alternative splicing, a longer ‘a’ form and a shorter ‘b’ form. It is not at all clear why the shorter desmocollin ‘b’ proteins exist, or indeed why multiple desmocollin and desmoglein genes are required. The desmosomal cadherins show tissue-specific patterns of expression with Dsc2 and Dsg2 ubiquitously expressed in tissues that produce desmosomes, and the other desmosomal cadherins largely restricted to stratified epithelial tissues where they exhibit differentiation-specific patterns of expression.

### Armadillo family

Armadillo proteins found in desmosomes include plakoglobin (*γ*-catenin) and plakophilins. Plakoglobin is a cytoplasmic protein that interacts with desmosomal cadherins, plakophilins and desmoplakin (see below). It is found both in desmosomes and adherens junctions where it is interchangeable with another armadillo protein, *β*-catenin. In addition to having an important structural role in cell junctions, plakoglobin acts as a signalling molecule (see below). Plakophilins 1, 2 and 3 (PKP1–3) show complex tissue-specific patterns of expression and dual localisation in desmosomes and in the nucleus. The details of their structural role in desmosomes have yet to be worked out, but it may be that the plakophilins are required to recruit desmoplakin to the plasma membrane (Getsios *et al*, 2004).

### Plakin family

Several plakin proteins, including desmoplakin, plectin, envoplakin and periplakin, localise to desmosomes. Of these, only desmoplakin is obligatory for normal desmosomal adhesion. Two isoforms of desmoplakin that are generated by alternative splicing of a single transcript are known. These differ only in the length of a central coiled-coil rod domain that separates N- and C-terminal globular domains. It is now well established that the N-terminal domain of desmoplakin binds to plakoglobin and plakophilin, whereas its C-terminal domain interacts with intermediate filaments (Getsios *et al*, 2004).

## PLAKOGLOBIN AND WNT/*β*-CATENIN SIGNALLING

Desmosomes are not just static structural entities and many now think of them as signalling centres. One of the ways that they could modulate intracellular signal transduction is by regulating the availability of plakoglobin. Plakoglobin is closely related to *β*-catenin, an important player in the Wnt/*β*-catenin pathway, and both interact with many of the same molecules. In the canonical pathway cytoplasmic *β*-catenin is phosphorylated by glycogen synthase kinase 3*β* and targeted for proteosomal degradation in the absence of a Wnt signal. Interaction of Wnts with their receptors block phosphorylation of *β*-catenin and allow it to accumulate in the cytoplasm and translocate to the nucleus, where it converts DNA binding proteins of the T-cell factor (Tcf)/lymphoid enhancer factor (Lef) family into transcriptional activators. It is now well established that accumulation and improper activation of transcriptional targets as a result of a failure to degrade cytoplasmic *β*-catenin is associated with tumours in a number of different tissues. More details of the Wnt/*β*-catenin signalling pathway can be found elsewhere (Li *et al*, 2006).

The role of plakoglobin in the Wnt/*β*-catenin pathway is not as defined as that of *β*-catenin. Like *β*-catenin, overexpression of plakoglobin is able to induce anterior axis duplication in *Xenopus* (Karnovsky and Klymkowsky, 1995) suggesting that it has a *β*-catenin-like signalling activity. However, expression of membrane-anchored forms of plakoglobin that show an exclusively cytoplasmic localisation and are unable to enter the nucleus also results in axis duplication (Merriam *et al*, 1997). This has led to the suggestion that plakoglobin stimulates Wnt/*β*-catenin signalling indirectly by blocking cytoplasmic degradation of *β*-catenin and allowing its translocation to the nucleus. Topflash is a plasmid that is used widely to measure Wnt/*β*-catenin signalling. It contains three Tcf binding sites upstream of a minimal promoter and luciferase reporter gene, and in many cases the increased Topflash activity that has been observed in response to overexpression of plakoglobin in transfected cells could be as a result of modifying *β*-catenin degradation and/or subcellular localisation. Plakoglobin can activate Topflash (all be it to a lesser extent than *β*-catenin), and the cyclin D1 and Nr-CAM promoters, in *β*-catenin-null embryonic stem cells showing that in some circumstances plakoglobin can act as an activator of Wnt/*β*-catenin signalling in its own right (as opposed to having an indirect effect via *β*-catenin) (Conacci-Sorrell *et al*, 2002).

It may be that plakoglobin is actually a *negative* regulator of Wnt/*β*-catenin signalling. Plakoglobin and *β*-catenin bind to adjacent sites on Tcf-4 and plakoglobin inhibits binding of Tcf-4 to DNA (Miravet *et al*, 2002). Thus, plakoglobin could reduce transcription of Wnt target genes by binding to Tcfs. In support of this idea, suppression of desmoplakin expression in cardiac myocytes (using small interfering RNA) leads to nuclear localisation of plakoglobin and suppression of Wnt/*β*-catenin signalling. This in turn leads to a transcriptional switch from myogenesis to adipogenesis and accumulation of fat droplets (Garcia-Gras *et al*, 2006). Conversely, phosphorylation of Tyr549 by the tyrosine kinase Fer results in increased binding of plakoglobin to adherens junctions and upregulation of Topflash activity (Miravet *et al*, 2003).

## PLAKOGLOBIN AND CANCER

Plakoglobin appears to have both positive and negative effects on cell growth. Thus, plakoglobin overexpression in transformed rat kidney epithelial cells promotes unregulated growth and foci formation (Kolligs *et al*, 2000). Unlike *β*-catenin, plakoglobin is a strong activator of *c-myc*, which is thought to be essential for plakoglobin's transforming ability. Another suggestion is that plakoglobin causes unregulated growth and foci formation (in human squamous carcinoma cells), as a result of induction of the pro-survival gene Bcl-2 and inhibition of apoptosis (Hakimelahi *et al*, 2000). In support of the latter idea, mutations within or near to a glycogen synthase kinase-3*β* consensus phosphorylation site have been discovered in advanced hormone refractory prostate cancer, and these coincide with strong nuclear accumulation of plakoglobin and a concomitant increase in Bcl-2 (Shiina *et al*, 2005). By contrast, in early prostate cancer, it appears to be loss of expression of plakoglobin, as a result of loss of heterozygosity (LOH) and hypermethylation of the plakoglobin promoter, that is important (Shiina *et al*, 2005). Indeed, the majority of studies suggest that plakoglobin has a tumour suppressor role. The plakoglobin gene is subjected to LOH in breast and ovarian cancers (Aberle *et al*, 1995) and loss of expression of plakoglobin has been correlated with poor clinical outcome in a number of cancers including non-small cell lung cancer (Winn *et al*, 2002). Plakoglobin is a histone deacetylase target gene and histone deacetylase inhibitors induce expression of plakoglobin in bladder carcinoma cells (Canes *et al*, 2005).

Experiments carried out both *in vivo* and *in vitro* have provided further evidence that plakoglobin suppresses proliferation. Transfection of plakoglobin into bladder cancer cell lines that show reduced levels of the protein reduces migration and suppresses the ability of the cells to produce tumours in nude mice (Rieger-Christ *et al*, 2005). Similarly, re-expression of plakoglobin in lung cancer cell lines inhibits cell growth on plastic and anchorage-independent growth in agar (Winn *et al*, 2002).

Overall, the evidence clearly supports a role for plakoglobin in cancer. Whether this is related to its participation in desmosomes remains unclear. It may be that desmosomes are able to sequester plakoglobin and so regulate its signalling activity in the same way that adherens junctions are apparently able to modulate *β*-catenin signalling (Gottardi *et al*, 2001; Kuphal and Behrens, 2006), but this is by no means is certain. Clearly, more work needs to be carried out to address this possibility.

## OTHER DESMOSOMAL CONSTITUENTS, WNT/*β*-CATENIN SIGNALLING AND CANCER

Many reports in the pathology literature have documented alterations in the expression of desmosomal cadherins during tumourigenesis, and LOH in the region of the desmosomal cadherin gene cluster on chromosome 18 has been observed in oesophageal and head and neck cancer (reviewed by Chidgey, 2002). In some cases, the immunohistochemistry data are contradictory and this may be at least in part due to the use of antibodies in early studies that do not distinguish between the various desmocollin and desmoglein gene products. Recent studies have shown loss of Dsg2 in gastric cancer (Biedermann *et al*, 2005; Yashiro *et al*, 2006), Dsc2 in colorectal cancer (Khan *et al*, 2006) and Dsc3 in breast cancer (as a result of promoter hypermethylation) (Oshiro *et al*, 2005). By contrast, Dsg2 and Dsg3 are overexpressed in squamous cell cancer of the skin (Kurzen *et al*, 2003) and head and neck cancer (Chen *et al*, 2007), respectively.

**Figure 1:**
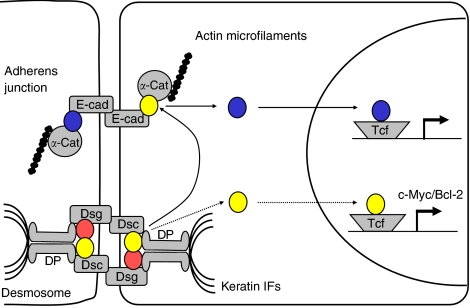
A model outlining how desmosomes could contribute to tumourigenesis. Unbroken arrows indicate that plakoglobin is released from desmosomes as a result of either loss or modulation of expression of desmosomal cadherins or desmoplakin and displaces *β*-catenin from adherens junctions. The latter translocates to the nucleus, stimulates transcription of *β*-catenin-responsive genes and ultimately results in cell proliferation. Broken arrows indicate that plakoglobin liberated from desmosomes translocates to the nucleus, stimulates transcription of genes, such as *c-myc* or Bcl-2, and promotes uncontrolled cell growth. Further possibilities (data not shown) are that loss of expression of plakoglobin itself could predispose to cancer by reducing its ability to antagonise *β*-catenin-mediated Wnt signalling or that plakophilins are involved (see text). Plakoglobin (yellow), *β*-catenin (blue), plakophilin (red). *α*-cat, *α*-catenin; DP, desmoplakin; E-cad, E-cadherin; IFs, intermediate filaments.

Alterations in the expression patterns of desmosomal cadherins in cancer could result in the release of plakoglobin from desmosomes, subsequent displacement of *β*-catenin from adherens junctions and increased Wnt/*β*-catenin signalling (Figure 1). Support for this idea has come from experiments in transgenic mice that are either null for Dsc1 (Merritt *et al*, unpublished) or overexpress Dsc3 in upper layers of the epidermis (it is normally expressed in basal layers) (Hardman *et al*, 2005). The mice exhibit hyperproliferation in the epidermis, and a redistribution of *β*-catenin from the membrane to the nucleus occurs in keratinocytes. Furthermore, cultured skin cells exhibit enhanced Topflash activity. It is perhaps surprising that Wnt/*β*-catenin signalling is enhanced in both experimental situations. Nevertheless, these results raise the possibility that modulation of desmosomal cadherin expression in cancer could stimulate transcription of *β*-catenin target genes. There is another possible outcome of changes in desmosomal cadherin expression patterns; release of plakoglobin from desmosomes could result in its translocation to the nucleus (Figure 1). Whether this would promote proliferation (by stimulating transcription of genes such as *c-myc* and Bcl-2) or have the opposite effect (by acting as a negative regulator of Wnt/*β*-catenin signalling) remains uncertain.

Overexpression of either Dsg2 or Dsg3 in suprabasal layers of mouse epidermis also results in keratinocyte hyperproliferation (Merritt *et al*, 2002; Brennan *et al*, 2007). In the case of Dsg2, precancerous papillomas appear in the skin of transgenic animals. Moreover, enhanced activation of multiple growth and survival pathways, including the phosphatidylinositol 3-kinase, mitogen-activated protein kinase, STAT3 and NF-*κ*B pathways, is observed (Brennan *et al*, 2007). In humans, patients with Dsg1 haploinsufficiency exhibit thickening of the skin on palms and soles, presumably as a result of defective adhesion and compensatory changes in keratinocyte proliferation and differentiation (Hunt *et al*, 2001). Overall, these finding indicate that alterations in desmosomal cadherin expression patterns, perhaps through modified intracellular signalling and/or changes in adhesive strength, has fundamental effects on cell behaviour, and can in some situations drive proliferation.

Alterations in plakophilins have been reported in cancer. PKP3 expression is lost at the invasive front of colorectal cancers (Aigner *et al*, 2007). By contrast, its expression is elevated in lung cancer and overexpression of exogenous PKP3 in COS-7 cells enhances growth and motility (Furukawa *et al*, 2005). Loss of expression of desmoplakin has been correlated with the progression of several cancers (Chidgey, 2002). PKP2 is able to stimulate Topflash activity (Chen *et al*, 2002), whereas loss of desmoplakin can lead to reduced Topflash activity (following translocation of plakoglobin to the nucleus) (Garcia-Gras *et al*, 2006). It appears that modulation of expression of all desmosomal constituents, including desmosomal cadherins, plakoglobin, plakophilins and desmoplakin, may be able in one way or another to influence Wnt/*β*-catenin signalling.

## A POTENTIAL ROLE FOR DESMOSOMES IN RHO SIGNALLING

Desmosomes may affect other signalling pathways. The protein p0071 (also known as PKP4) exhibits dual localisation in desmosomes and adherens junctions, at least in some cell types (Hatzfeld *et al*, 2003). It shares significant sequence homology with PKPs1–3, but is more closely related to the adherens junction protein p120-catenin. The role of p120-catenin in modulating the activity of Rho GTPases, as well as regulating cadherin turnover at the membrane, is well established (Reynolds and Roczniak-Ferguson, 2004). Recent data have now shown that p0071 is also able to regulate Rho signalling, and it has been proposed that p120-catenin and related proteins may act as scaffolding proteins that stabilise Rho signalling complexes (Wolf *et al*, 2006). Rho GTPases, which act as molecular switches that control diverse functions of the cell, have been implicated in tumourigenesis (Gomez del Pulgar *et al*, 2005), and the exciting possibility that desmosomes (via PKPs 1–3 and p0071) could modify their activity deserves further study.

## DESMOSOMES AND THE EPITHELIAL–MESENCHYMAL TRANSITION

**Figure 2:**
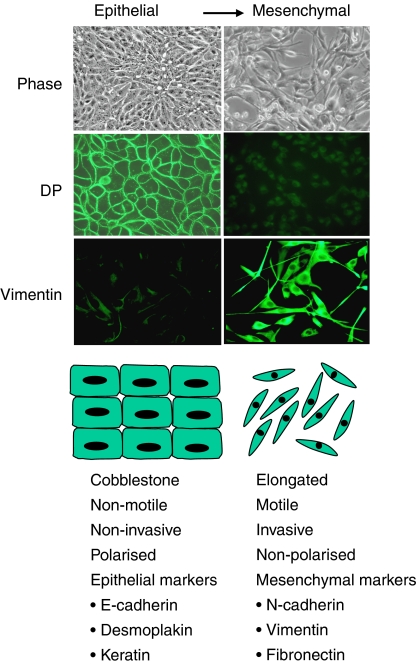
Epithelial–mesenchymal transition. Epithelial–mesenchymal transition in Madin–Darby canine kidney epithelial cells is characterised by a change in morphology, loss of expression of desmoplakin and gain in expression of vimentin. DP, desmoplakin.

Desmosomes may have an impact on tumourigenesis through their ability to modify signalling by armadillo proteins (see Figure 1). They may also be important in cancer progression as a consequence of their role in the epithelial–mesenchymal transition (EMT). Epithelial–mesenchymal transition is an indispensable mechanism for morphogenesis during embryonic development, and is implicated in wound healing and conversion of early-stage tumours into invasive cancers. During EMT, epithelial cells undergo changes in morphology and acquire the migratory and invasive characteristics of mesenchymal cells (Figure 2). Disruption of both adherens junctions and desmosomes must occur for epithelial cells to dissociate during EMT. A hallmark of EMT in cancer is loss of expression of the adherens junctions component E-cadherin, which can occur as a result of genetic and/or epigenetic mechanisms. Mutations in desmosomal cadherins have yet to be found in cancer, but loss of expression of desmosomal cadherins has been documented in a number of cases (see above).

The closely related zinc-finger transcription factors Snail and Slug are key EMT regulators that repress E-cadherin expression during development and cancer. Snail, Slug and the more distantly related zinc-finger proteins ZEB1 (*δ*EF1) and ZEB2 (SIP1), which are also strong repressors of E-cadherin, bind to E-box motifs in the E-cadherin promoter. Transfection of Snail, Slug and ZEB2 into cultured epithelial cells results in the dissolution of desmosomes and EMT (e.g., Vandewalle *et al*, 2005). It may be that desmosome dissolution occurs simply as a secondary consequence of repression of E-cadherin. However, recent data have suggested that Slug, but not Snail, plays an important role in re-epithelialisation by disrupting desmosomes but not adherens junctions at wound margins (Savagner *et al*, 2005). Furthermore, several desmosomal genes have E-boxes in their promoters and ZEBs 1 and 2 are able to downregulate expression of PKP2 and PKP3, respectively, by binding to their promoters (Vandewalle *et al*, 2005; Aigner *et al*, 2007). Thus, it is entirely possible that Snail and related proteins play an active role in loss of expression of desmosomal components.

Although by no means definitive, there is some evidence that suggests that desmosome assembly may cause cells to undergo EMT reversal, the so-called ‘mesenchymal–epithelial conversion’. Treatment of squamous cell cancer cells with inhibitors that block the epidermal growth factor receptor and prevent tyrosine phosphorylation of desmosomal constituents promotes desmosome assembly (but has no effect on E-cadherin expression and solubility), and this is coincident with a change in morphology from a fibroblastic to epithelial appearance (Lorch *et al*, 2004). Introduction of plakoglobin into SCC9 cells, which are deficient in both plakoglobin and E-cadherin, has a similar effect (Parker *et al*, 1998). Transfection of a desmocollin, a desmoglein and plakoglobin into fibroblasts is insufficient to produce bona fide desmosomes and does not elicit a change in morphology, but is enough to inhibit invasive behaviour (Tselepis *et al*, 1998).

## CONCLUSION

Definitive evidence that links desmosomes to cancer is still lacking. However, a substantial amount of evidence is available that supports the idea that they are involved in progression of the disease. Changes in expression of desmosomal constituents have been documented, and mutations in the plakoglobin gene have been linked to the pathogenesis of prostate cancer. More studies that comprehensively document changes in desmosomal constituents during tumourigenesis are required and future challenges will include identifying mutations (if any) in other desmosomal constituents. Defining the role of desmosomes as mediators of intracellular signal transduction and identifying changes in signalling pathways that occur in response to alterations in the normal patterns of expression of desmosomal constituents will be an important step forward. Understanding the mechanisms that result in desmosome disruption during EMT will advance our understanding of this critical cell biological process. Progress in these areas will lead to a better understanding of the role of desmosomes in normal tissue homeostasis and malignancy.
